# NeuroTerrain – a client-server system for browsing 3D biomedical image data sets

**DOI:** 10.1186/1471-2105-8-40

**Published:** 2007-02-05

**Authors:** Carl Gustafson, William J Bug, Jonathan Nissanov

**Affiliations:** 1 Laboratory for Bioimaging and Anatomical Informatics, Department of Neurobiology and Anatomy, Drexel University College of Medicine, 2900 Queen Lane, Philadelphia, PA, 19129 USA

## Abstract

**Background:**

Three dimensional biomedical image sets are becoming ubiquitous, along with the canonical atlases providing the necessary spatial context for analysis. To make full use of these 3D image sets, one must be able to present views for 2D display, either surface renderings or 2D cross-sections through the data. Typical display software is limited to presentations along one of the three orthogonal anatomical axes (coronal, horizontal, or sagittal). However, data sets precisely oriented along the major axes are rare. To make fullest use of these datasets, one must reasonably match the atlas' orientation; this involves resampling the atlas in planes matched to the data set. Traditionally, this requires the atlas and browser reside on the user's desktop; unfortunately, in addition to being monolithic programs, these tools often require substantial local resources. In this article, we describe a network-capable, client-server framework to slice and visualize 3D atlases at off-axis angles, along with an open client architecture and development kit to support integration into complex data analysis environments.

**Results:**

Here we describe the basic architecture of a client-server 3D visualization system, consisting of a thin Java client built on a development kit, and a computationally robust, high-performance server written in ANSI C++. The Java client components (NetOStat) support arbitrary-angle viewing and run on readily available desktop computers running Mac OS X, Windows XP, or Linux as a downloadable Java Application. Using the NeuroTerrain Software Development Kit (*NT-SDK*), sophisticated atlas browsing can be added to any Java-compatible application requiring as little as 50 lines of Java glue code, thus making it eminently re-useable and much more accessible to programmers building more complex, biomedical data analysis tools. The *NT-SDK *separates the interactive GUI components from the server control and monitoring, so as to support development of non-interactive applications. The server implementation takes full advantage of data center's high-performance hardware, where it can be co-localized with centrally-located, 3D dataset repositories, extending access to the researcher community throughout the Internet.

**Conclusion:**

The combination of an optimized server and modular, platform-independent client provides an ideal environment for viewing complex 3D biomedical datasets, taking full advantage of high-performance servers to prepare images and subsets of associated meta-data for viewing, as well as the graphical capabilities in Java to actually display the data.

## Background

Volumetric anatomical atlases have become more prevalent as desktop computers have grown more powerful and have been provided with greater storage capacity. Digital atlases can be superior to print atlases in a number of ways: true 3D atlases are not restricted to just the limited number of sectional views typically presented in a print atlas but can be resliced in any arbitrary orientation [1]; the user can create spatially coded annotations or segmentation regions to share with other researchers [1]; they can be used as a canonical framework to support semi- or fully-automatic atlas-guided segmentation [2,3]. Furthermore, experimental datasets can be aligned to the atlas and be synchronously navigated, showing equivalent cross-sectional planes across all datasets. Lastly, digital atlases provide a universal reference frame for other biomedical image or otherwise spatially coded data sets, enabling correlated analysis of complementary data modalities within an equivalent geometric scaffold [4,5]. For example, the system described here is being employed to spatially index The Mouse Brain Library [2] and is being employed within the Mouse BIRN Integrated Atlasing Tool (MBAT) associated with the Biomedical Informatics Research Network (BIRN)[5,6].

A number of digital atlases are now available, for several species and imaging modalities. Focussing specifically on the nervous system of the C57BL/6J inbred strain of *Mus musculus *(house mouse), a controlled genetic population critical for genetic and functional genomic research, there are several existing brain atlases: the NeuroTerrain[1] and the SMART (Spatial Markup and Rendering Tool) Atlas developed for use within BIRN[5] are Nissl-based light-microscopic atlases most closely analogous to the classical print, Nissl-stained histological mouse brain atlases in wide use[7,8]; UCLA's Laboratory for Neuro Imaging (LONI) Mouse Atlas Project (MAP) includes Magnetic Resonance Microscopy (MRM) image sets, as well as basic histochemical and immunohistochemical tissue sets[9]; Ma et al also described an MRM mouse brain atlas[10]. The Edinburgh Mouse Atlas Project (EMAP) [11] includes classic histochemical microscopy, Confocal Laser Scanning Microscopy (CLSM) and Optical Projection Tomography (OPT) image sets captured at multiple spatial resolutions from various developmental stages, though providing only limited nervous-system specific morphological detail.

Several large-scale, mouse brain-specific gene expression reference data sets are being constructed which are critical resources for atlas-based integrated analysis of neurodevelopment and neurodegenerative disease [12]. All of these data sets are anatomically-mapped, some by virtue of being assays performed directly in a tissue context both using standard in situ immunohistochemistry, either fluorescence [13,14], alkaline-phosphatase [15], digoxygenin-labeled antisense riboprobes (The Allen Brain Atlas)[12], or radioactivity (Brain Gene Expression Map or BGEM) [16], as well as GFP-reporter constructs used in the Gene Expression Nervous System Atlas (GENSAT) BAC Transgenics project [17]. For others, the anatomical context is defined only to a relatively coarse level of granularity by microdissecting mesoscopic brain regions [18-20].

Associated with these brain atlas projects and reference data sets are software systems for browsing the images in a 3D context (see Table 1), though only a subset of these supports reslicing the 3D data sets to produce novel 2D views independent of the orientation in which the 2D images were originally acquired (e.g., the NeuroTerrain Client-Server Atlas system described here, MAP's Synchronized Histological Image Viewing Architecture (SHIVA)[9] browser, the EMAP JAtlasView[21], the Mouse Brain Image Visualizer (MBIV)[22] used by Ma et al.). Though sharing many functional features common to 3D anatomical analysis systems, each of these systems provides many unique features. Many of these atlas software systems are designed to run on local desktop computers; this, unfortunately, restricts their use to fairly powerful computers, since these multi-gigabytes atlas data sets require substantial computing power to be manipulated interactively.

**Table 1:** 3D Data Browsers Used For Neuroanatomical Atlas Viewing

**Viewer Application Name**	***3D Slicer***
**Viewer Developer Laboratory**	Surgical Planning Laboratory, Brigham and Women's Hospital, Boston, MA, USA and The M.I.T. Computer Science and Artificial Intelligence Lab, M.I.T., Cambridge, MA, USA
**Abbreviation**	n.a.
**Viewer URL**	
**Software Reference**	[29]
**Exemplar Neuroimaging Project**	Psychiatric NeuroImaging Lab, Brigham and Women's Hospital, Harvard Medical School, Boston, MA, USA
**Atlas URL**	
	
**Viewer Application Name**	***JAtlasViewer***
**Viewer Developer Laboratory**	MRC Human Genetics Unit, Western General Hospital, Edinburgh, UK and The Institute of Human Genetics, University of Newcastle, Newcastle-upon-Tyne, UK
**Abbreviation**	n.a.
**Viewer URL**	
**Software Reference**	[21]
**Exemplar Neuroimaging Project**	The Edinburgh Mouse Atlas Project (EMAP), Human Genetics Unit, MRC and The Section in Biomedical Sciences/Division of Biomedical and Clinical Sciences, The University of Edinburgh, Edinburgh, UK
**Atlas URL**	
	
**Viewer Application Name**	***The Mouse Brain Image Visualizer***
**Viewer Developer Laboratory**	Center for Translational Neuroimaging and Computational Science Center, Brookhaven National Lab, Brookhaven, NY, USA
**Abbreviation**	MBIV
**Viewer URL**	
**Software Reference**	[22]
**Exemplar Neuroimaging Project**	3D MRI Digital Atlas Database of Adult C57BL/6J Mouse Brain, Center for Translational Neuroimaging, Brookhaven National Lab, Brookhaven, NY, USA
**Atlas URL**	
	
**Viewer Application Name**	***NeuroTerrain Client-Server Atlas System***
**Viewer Developer Laboratory**	Laboratory for Bioimaging and Anatomical Informatics, Dept. of Neurobiology and Anatomy, Drexel University College of Medicine, Philadelphia, PA, USA
**Abbreviation**	NetOStat/NT-SDK
**Viewer URL**	
**Software Reference**	This report
**Exemplar Neuroimaging Project**	The Mouse Brain Library, Neurogenetics at University of Tennessee Health Science Center, Memphis, TN, USA
**Atlas URL**	
	
**Viewer Application Name**	***The Pittsburgh Supercomputing Center Volume Browser***
**Viewer Developer Laboratory**	Pittsburgh Supercomputing Center and the PSC Biomedical Initiative, The Pittsburgh Supercomputing Center, Carnegie Mellon University and the University of Pittsburgh, Pittsburgh, PA, USA
**Abbreviation**	PSC Volume Browser
**Viewer URL**	
**Software Reference**	[23]
**Exemplar Neuroimaging Project**	Variational Mouse Brain Atlas, Mouse Imaging Center, Toronto Centre for Phenogenomics, Mount Sinai Hospital, The Hospital for Sick Children, University Health Network and St. Michael's Hospital, Toronto, Ontario, CA
**Atlas URL**	
	
**Viewer Application Name**	***Synchronized Histological Image Viewing Architecture***
**Viewer Developer Laboratory**	The Laboratory of Neuro Imaging (LONI), Department of Neurology, UCLA, Los Angeles, CA, USA
**Abbreviation**	SHIVA
**Viewer URL**	
**Software Reference**	[9]
**Exemplar Neuroimaging Project**	The Laboratory of NeuroImaging (LONI) Mouse Atlas Project (MAP), UCLA, Los Angeles, CA, USA
**Atlas URL**	

This list of available 3D image set browsers currently used to manipulate mouse brain atlas data sets, provides links both to the developers of the software tool, as well as to a representative neuroimaging project for which that browser is currently being employed. This is a subset of the available 3D anatomical browsers used in all species, a list of which can be obtained from the Internet Analysis Tools Registry maintained Dr. David Kennedy at the MGH, Boston MA [30]. By our count, there are currently 38 3D Volume data set viewers available for use.

The amount of data processing required also militates against most common cross-platform approaches to browser design, since many of these approaches depend on relatively slow byte-code interpreters. One can carefully design viewers to work around some of the memory and performance limitations of typical desktop computers to create hybrid cross-platform atlas browsers [21], but our experience has shown a client-server implementation is typically easier to extend and keep inter-operative. This architecture reduces the processing and memory load on the local desktop, shifting it to a server shared among many clients. Proper server architecture design can enable the system to scale its performance to synchronously serve multiple 3D atlas data sets to many simultaneous users. The use of a client-server architecture also simplifies version management, since server-side enhancements need only be added to just a few systems in known locations, and client upgrades can be provided easily via web distribution. Finally through the use of a well-thought-out client design, data from many sources can be integrated to accommodate specific research needs, greatly magnifying the power of the system.

A number of the above-referenced atlas-based 3D anatomical analysis systems are network based, but they all have practical limitations. SMART Atlas, for example, is not built on a true 3D dataset providing only those views available in the Franklin & Paxinos published atlas [7]. SHIVA from the LONI MAP group and MBIV used by Ma et al. each allows the user to slice a 3D set but only on planes orthogonal to one of the 3 standard anatomical orientations (coronal, sagittal & horizontal), not off-axis at an arbitrary angle which is often required in the course of a study. JAtlasView from EMAP does allow for arbitrary-angle slicing, but, as with SHIVA and MBIV, you must download the entire 3D data set you intend to visualize to your local machine and require special knowledge to properly install the sets. In addition, all of these implementations exist as islands in the Internet, with no real connections to other data sources. Only the PSC Volume Browser [23] used to view data from Siddiqui, et al. is fully implemented as a client-server architecture. Though the powerful PSC Volume Browser architecture is modular, it was not designed to be integrated as an element within a larger analysis framework, neither as a Graphical User Interface (GUI) component nor as a middleware connectivity Application Programming Interface (API). The JAtlasViewer does promote use of it's various components for construction of more complex applications and, in particular, provide useful wrappers for encapsulating new compiled functionality; however, it was not specifically designed as a development kit, so a significant amount of learning and additional coding is required to properly use these modules. In this paper we will describe a full 3D slicing server implementation supporting arbitrary resampling both on and off the traditional axes and the *NT-SDK *with an open API for easy integration into other Java-based biomedical image visualization/analysis environments.

## Implementation

The client-server architecture is a well known solution to the need for providing access by multiple users to a single, controlled data source. Presented here is a basic server design, a client development kit, and the language they use to communicate. The server architecture is highly modular, allowing individual server-side components to be enhanced or replaced without affecting the other components or existing client implementations. The NeuroTerrain Atlas Server (*NtAS*) design is multi-threaded to take advantage of multi-core multi-processor systems and provide enhanced scalability. It allows for remote administration using standardized XML-based communications. The NetOStat client [see Additional files 1 and 2] is implemented directly on top of the *NT-SDK, a framework *promoting integration into existing applications and providing access to the *NtAS *with minimal coding. The *NT-SDK *Java framework supports both end-user client functionality, displaying atlas data graphically, as well as middleware and backend uses, supporting integration of data sets hosted by the *NtAS *and image file repository (i.e., image sets aligned to a NeuroTerrain atlas) into a data federation framework, for example[3]. These two distinct features of the SDK result from it being designed as two separate libraries – NT-SDK-NtAS-comm.jar and NT-SDK-NetOStat-GUI.jar. The latter depends on use of the former, whereas middleware can utilize the former without need of the end-user interactive GUI components.

### Main Server Modules

There are five main modules included in the server. These are the Server Manager (SM), the Data Manager (DM), the Slicers, the Processing Queue Manager (PQM) and the modules handling the two public network interfaces. The servers, slicers, and server manager each run in their own threads; the others either run in their client connection's threads or spawn additional threads as needed.

#### Server manager

The server manager is responsible for the overall operation of the server application:

1. exposing a control socket for administration;

2. loading or unloading datasets;

3. associating datasets with ontologies;

4. managing user (client) access;

5. managing the properties of the servers; and,

6. starting/stopping multiple instances of the slicer threads as needed.

Administrative clients communicate with this module via a standardized XML stream over a TCP socket. Secuity can be handled using SSL, and/or control limited to a UNIX-domain socket, which restricts access to processes running on the local host.

#### Servers

This NtAS implementation actually consists of two linked servers, each with its own communication socket. One server implements a binary interface, used to retrieve sliced images of the dataset; in the other, XML data is used to return single-instance data items.

The binary server is designed to maintain a persistent TCP session connection, similar to the way telnet is implemented. Once the client logs in, a data structure maintaining atlas data set state is created and linked to the user via the same TCP socket (IP number/port number combination) used to connect to the server. Once the DM identifies the client and approves a connection, a list of atlases/datasets available for browsing is returned. The client requests the desired atlas, and the server returns the dimensions and resolution of the chosen dataset. Subsequent session communication between the client and the server include serialized requests for slice (image) data based on the client's specified plane orientation, and requests to show/hide any available segmentation, including presentation metadata.

**Figure 1:**
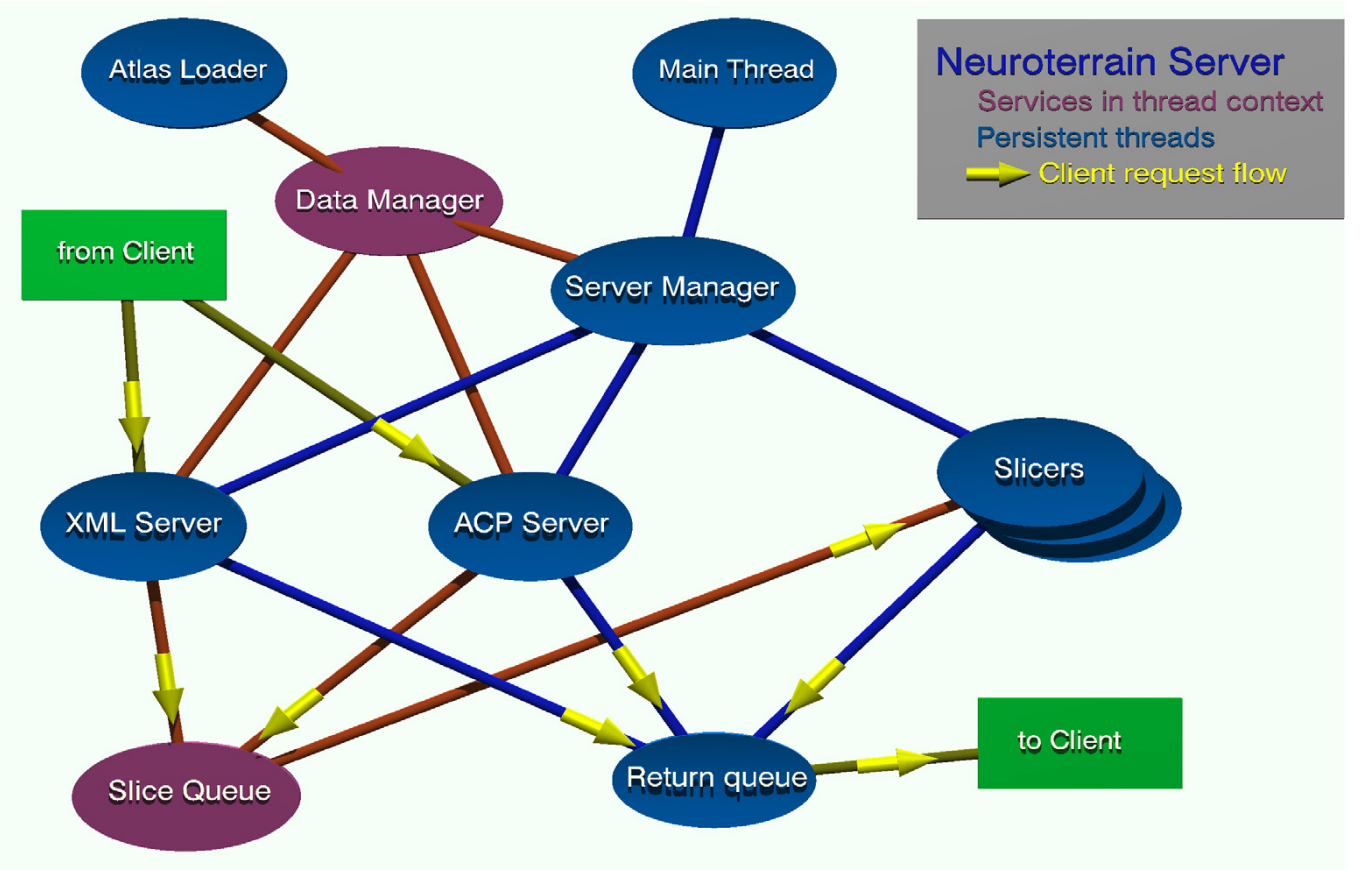
**NeuroTerrain Server Functional components**. The NeuroTerrain Server is composed of several distinct software modules which inter-relate in a precise way, a subset of which interact directly with a connected client. Please see Main Server Modules section of the text for a more complete description of these separate components.

The XML server uses a single-transaction oriented protocol much like http. The user logs in, is approved, and can then request information about the selected atlas, including 2D paths representing the intersection of a segmented volume and a specified slicing plane, etc. Per-client state data is tracked via a unique session ID which expires after a period of inactivity. This interface is an implementation of an inter-atlas interoperability standard developed within the Mouse BIRN subproject of BIRN[5] which was designed to promote atlas data sharing amoungst the Mouse BIRN neuroanatomical data management clients NeuroTerrain, SMART Atlas and SHIVA. Figure 1 provides an architecture diagram to support the description given below.

#### Slicers

The data slicer runs in an independent thread, and accepts slicing requests from a queue fed by the servers. There is no theoretical limit to the number of instantiated slicing threads, but there is generally no practical advantage to having more slicing threads than processor cores on the host computer. Each slicer thread is able to handle both image pixel requests from the binary server and segmentation path requests from the XML server. Results of the slicing operation are returned to the client by the slicer via a return queue.

The key to the real-time responsiveness of the Slicer component is the use of the Macrovoxel 3D data format which provides for an extremely fast nearest-neighbor-based assembly of any arbitrary place on or off the original imaging axis[1]. Both the pixel data sets as well as the associated segmented Volumes of Interest (VOI) are stored in an efficient format.

Binary slicing requests are serialized. This enables client to provide live navigation by tracking the mouse pointer, sending a continuing stream of slice requests as the mouse moves. A slicer thread checks each incoming slice request serial number against the current value in the client's state data; stale requests are dropped. Returned slices are also serialized; several slicing threads may be running, and two different threads may be processing requests from the same client. The client ignores stale slices returned. Region of Interest (ROI) path requests are not serialized, since the order in which they are returned is not critical to the way they are processed by the client and presented to the user. This enables the server to process ROI path requests more efficiently.

#### Data manager

The DM is responsible for loading, unloading and maintaining access to datasets, and associating ontologies with the datasets. It also manages user access permissions to these datasets.

#### Process queues

The process queues are used to communicate between the server threads and the slicer threads. Requests for slicing service are placed in a queue as a data structure including client ID and state information, target information, and requested action. Each slicer calls a PQM function that blocks waiting on a condition variable for queue state. Once a request is received, the slicer examines the request type, and dispatches it to the appropriate handler – binary or XML. This handler satisfies the request, and then returns the resulting data to the PQM return queue, where it is then forwarded to the client.

This module, implementing queues which the slicing thread feeds off, readily allows for multiple slicing threads, and also allows for threads to be started or stopped on the fly, if so desired, greatly enhancing the scalability of the server.

### Major Client Modules

The *NT-SDK *framework includes two major branches. The first encapsulates the API to communicate directly with the server providing an interface other software developers may use to obtain specific 2D slices and associated meta data (ROI paths, brain region ontological specifiers) directly from the *NtAS*.

The other library, contains GUI components, is end-user oriented to support a researcher interacting directly with the server. This later module uses the former to handle its communications with the server and is encapuslated in a pair Java JPanels for easy inclusion in other applications. The first class encapsulates the Virtual Knife navigator (*VK*, see below), while the other provides a viewer for a server-hosted data set. The core client connection controller has been implement to support instantiating the SV JPanel as many times as necessary to open multiple connections to a single atlas data set (e.g. linked coronal and sagittal views) or connections to multiple data sets. *VK *action drives sumultaneous navigation through all open views. The view classes can be created as JPanels for tight integration with the parent application, or as free-floating JFrames.

#### Server Interfaces

The NtAS interactions are encapsulated in the NT-SDK-NtAS-Comm framework. Within this library, the client communicates with the server via two discrete sub-interfaces. One, derived from the original *MacOStat server, transfer all data *in binary format – values are encoded as either 32 bit floats and integers or strings (atlas or region names, for example). Integer codes transmit NtAS request commands chosen to give recognizable display in a binary data stream. This interface provides efficient, dynamic image-oriented transactions: return of a sliced volume being the main example, but control of the *VK *is also included here. The binary commands of this socket protocol are implemented as a collection of Java classes handling all the communication details, making server easily available to external programmers by merely creating a new object of a particular command class and sending it to the communication manager's write command. A communication controller embedded in the NT to mediate server transactions,

The NT-SDK-NtAS-Comm also provides callbacks as a Java Interface to monitor NtAS responses to the binary commands – e.g., the latest slice image or associated metadata. By implementing these methods, an applications can dyanically react to new server responces as required.

The other request interface in the NT-SDK-NtAS-Comm library is based on XML-formatted text streams, and is used to poll atlas state: information such as the 2D planar location within a mounted data set, paths describing the intersection of VOIs with the VK, and so on.

Detailed specifications for these protocols can be found at the web site [24].

#### Graphical User Interface

**Figure 2:**
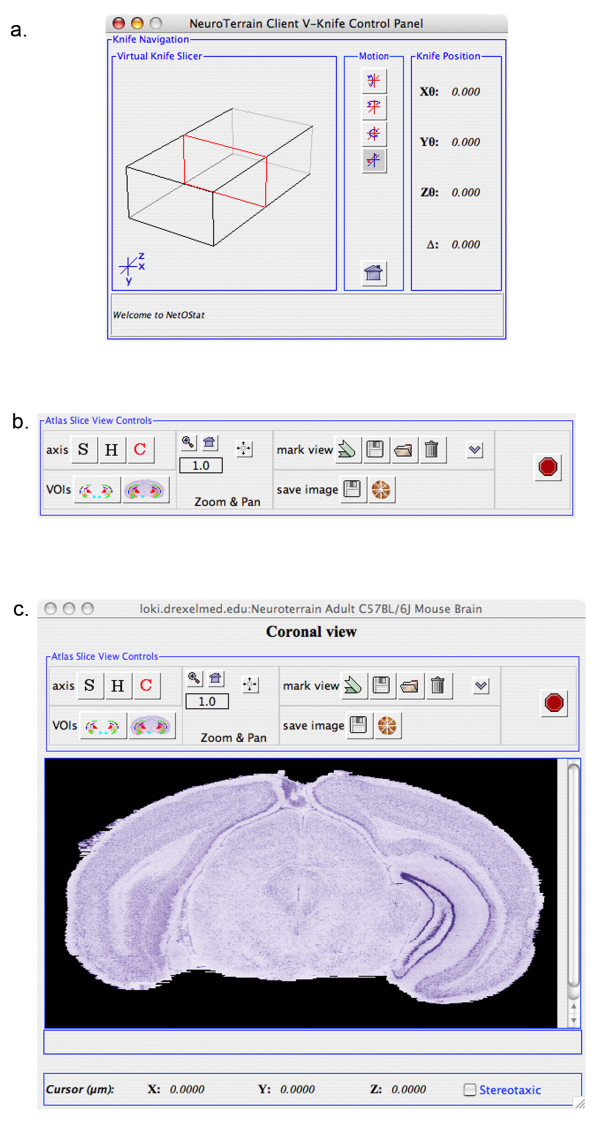
**NT-SDK User Interface components**. ***(a) ***Via the *Virtual Knife *wireframe, the user can continuously adjust the cross-sectional slice position through the current 3D atlas volume(s) mounted on the server. By dragging the mouse left or right over the wireframe, the knife is displaced along the currently selected anatomical axis. The *Knife Position *indicates the cross-section location in terms of the current linear displacement (Δ) along the slice axis, and the rotational angles about the standard X, Y, and Z cartesian axes. The knife can also be minimally nudged via the left and right arrow buttons. The *Motion buttons *specify whether mouse movement causes translation about this axis, or rotation about the X, Y or Z cartesian axes. ***(b) ***These SV control panel tool buttons alter the state of the data in the viewer. The *Axis *specifies whether the knife is adjusted along the coronal, sagittal, or horizontal cutting axes. The VOI buttons enable the investigator to toggle VOI viewing on/off and to choose the segmented regions to view. The *Zoom button *adjusts the scale of the current atlas view from 0.1× to 10.0×. When zoomed in, a crop window is imposed to limit network data transmission, which can be adjusted by dragging on the grayscale view or centered via the Center Crop button. The Mark buttons are used to save a specific view cross-section position for recall, and enable the investigator to respectively save, open, and clear the marks menu. The Save Image buttons respectively save the current slice view to a file or provide a means to resample an extended portion of the data set under view. ***(c) ***This full view of the *NtAS *Client (NetOStat), shows the relation of the controls in *b *to the gray-scale cross-sectional atlas view. At the bottom of this frame are controls to view the current image scale and cursor location, when the mouse cursor is placed over the atlas image. Mouse over location can be translated into stereotaxic coordinates when coordinate transformation matrices are available, as they are for the NT Adult Mouse Atlas.

The GUI consists of:

• the VK JPanel used to set the current 3D atlas cross-section view (Figure 2a);

• a set of related controls specifying how dragging of the mouse over the VK translates into movement of the knife (Figure 2a &2b);

• a Slice Viewer (SV) JPanel for each atlas data connection (Figure 2c);

• a collection of menu commands providing additional functionality (see below).

This interactive functionality is embedded in the NT-SDK-NetOStat-GUI framework. By obtaining these JPanels from this library, an enclosing application incoporates the entire binary server API, as well as the GUI controls listed above. Detailed specifications for the *NT-SDK *API can be found at[24].

The *knife *is presented as an intersecting plane within a wireframe rectangular volume whose dimensional extent is the x, y, z bounds of the 3D atlas dataset under view (Figure 2a). A series of tool buttons enables an user to specify how mouse dragging over the wireframe afects VK movement. The investigater may translate the knife along the slicing axis, or rotate it about any of the three cartesian axes. A series of buttons in the SV control panel enables the user to select the cutting axis to present slices along any one of the standard anatomical axes – coronal, horizontal, or sagittal (Figure 2b). Thus, a user can slice through the 3D data set in an arbitrary plane either on or off axis. Each user intiated VK movement triggers a request to the server resulting in an updated atlas image in the SV (Figure 2c). A client preference setting enables an investigator to adjust the scaling of mouse cursor movement to knife movement on the server (default = 5 server slice pixels/1 client cursor pixel).

**Figure 3:**
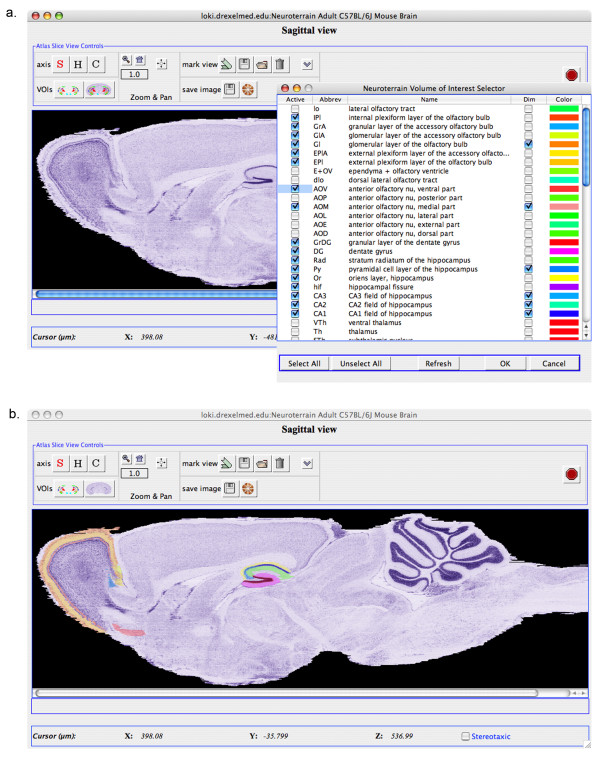
**Atlas VOI selection**. The VOI selection button sends a request to the server to obtain a list of VOIs for the currently loaded 3D atlas data set. This listing of VOIs is presented to the researcher on the above form ***(a)***, from which she can selectively activate specific VOIs for viewing superimposed on the atlas view. ***(b)***.

The *NT-SDK-NetOStat-GUI *also can provide the *VK and SV *interfaces as separate, floating frames and enable the researcher (or external SDK programmer) to create multiple *NtAS *connections. Each of these distinct SVs (Figure 3b) comes with a collection of tool buttons supporting easy view specific actions: switch slice axes, select VOIs, pan & zoom, save knife locations, and save atlas grayscale views. A single VK provides coupled navigation across all views (Figure 3a). This enables the researcher to simultaneously co-navigate multiple, aligned Macrovoxel data sets. From the point-of-view of external programmers, each SV is provided as a separate object not only providing access to the JFrame itself, but also the callbacks tied to each one (e.g., automatic updates to atlas slice axis, grayscale image, zoom level, etc.). All navigational interoperability amoungst the distinct SV connections and the *VK *JFrame is handled internal to the *NT-SDK*, though this activity can also be driven from the external program. The programmer can also request these views and the VK interface be passed as JPanels for tighter integration into the external program.

The multi-threaded nature of the server makes it possible to support these parallels data connections. Multiple NtAS instances can also be setup when user demand requires more slicing throughput.

A separate dialog allows the user to examine the list of volumes of interest or segmentated regions included in the atlas, select a subset for display and to specify a particular color for the volume (Figure 3a). These colored ROIs are superimposed on the main display either as a semi-opaque mask or as an outline (Figure 3b). A researcher can toggle individual ROIs on/off, or selectively dim certain ones to emphasize a selected region among a set of related regions.

A collection of hierarchical menus under the main *NeuroTerrain Atlas Server *menu supports the following, some of which are described in more detail in the following section on client-server interactions:

• login to and logout from and ping the server;

• select atlas data sets for viewing;

• test network throughput to a given NtAS.

These features are all described in more detail below.

### Client-Server Transactions

In this section, the various interactions between client and server are described. There are five basic transactions: Client login, selection of an atlas or dataset to browse/query, selection of an axis along which the VK is moved, knife positioning, and requests for 2D path cross-sections through segmented volumes of interest.

#### Login

Prior to performing any other action, the client must log in to the server. This allows the server to set up the necessary session structures, and registers the particular client implementation or user for any special actions desired. For example, at this point the user-agent name is transmitted by the client. On the server side, this can be used to select a particular default dataset, to specify that only a subset of metadata is returned to the client, or restrict the client to a subset of mounted data sets. The user agent is a configurable value in a Java properties file within the *NT-SDK*, so clients can be distributed to a user base with unique and defined set of default conditions the server can enforce. Using either the binary or XML API, the login message contains the user-agent ID, and optionally a user ID. Once the login message has been processed by either server protocol, an acknowledgment is returned. In the case of the binary API, the return message contains a list of all available 3D datasets (atlases or other Macrovoxel files), associated with a reference number. In the case of the XML interface, the returned message contains the unique session ID. A client logged in via the XML API can then send a query for a list of available datasets.

#### Atlas selection

Once successfully logged in via the binary API, the client further configures the session by choosing which available dataset to view. This results in the server returning a data block containing the dimensions and resolution of the requested dataset. When using the XML API, a client does not declare a specific atlas data set up front, as the desired atlas must be included in all subsequent requests.

#### Axis selection

After selecting an atlas, a client using the binary API must specify the axis along which the VK will be moved. This value defaults to the Z-axis, which provides the anatomically standard coronal tissue presentation The X-axis and Y-axis similarly provide sections in the sagittal and horizontal orientations, respectively. A coronal view of the NeuroTerrain mouse brain histological atlas is presented in Figure 2c.

#### Knife position change

Once atlas and axis selections have been completed, the system is ready to return sliced volume data. To initiate slice retrieval, the client sends a knife position request. This involves specifying a sequence number for serialization purposes, an offset along the slicing axis, and a triplet of angles describing the orientation of the VK to the slicing axis (angular rotation about the standard cartesian x, y, and z axes). The server queues this request for the slicer, along with client address information and the client sequence number. In turn, the slicer executes the slice requests, and returns pixel data as a series of linear pixel arrays or rasters, along with raster positioning information and the slicer's sequence number.

#### ROI request

A client using the XML API is able to request paths describing the intersection of VOIs with the VK. To do this, the client provides identifiers for the VOIs to be sliced, and the orientation of the VK. Presently, brain region names and abbreviations developed in our lab are hard-coded into a list and linked to the assocated VOI data set. We are in the process of incorporating a more dynamic means of associating anatomical segmented region identifers from a public knowledge source, such as NeuroNames[25] in the case of brain region volumes. A complete set of the available VOIs for the current atlas dataset on view may be obtained from the server. VOI slice requests are also queued for the slicer, and the 2D ROI cross-section(s) for the current knife position are returned in the form of an ordered set of cartesian coordinates. More specifically, individual ROIs are returned to the client as pointlist 3D objects where all points lie in a single plane.

#### Other operations

Several other operations are available. For example, the client can specify a magnification or zoom level for the returned slice images, currently spanning the range from 0.1× to 10×. To support dynamic response to knife movement at higher zoom, the view is cropped and the user can pan the cropping window across the entire extent of the data set. Server throughput and latency (*ping) operations provide a means for a researcher to assess the performance of their network connection from any point on the Internet*.

## Results and Discussion

The system described here was designed with flexibility in terms of functionality, and ease of use or implemenation for the client in mind.

### System flexibility

By settling on a client-server implementation, we are able to isolate the low-level implemenation details of the slicers, and image data structures from the high-level implemenation of the client. This means, for example, although the server currently sources image data from disk files, it is entirely possible to source all or part of the dataset from a database. This would allow individual users, for example, to specify their own segmentation to be served by this system. Making this a back-end function means that this enhancement may be provided to the client with no change needed to the client software.

Another change would be addition of, for example, the ability to serve datasets with higher resolution areas of interest, or to provide overlaid data from mutiple spectral channels.

Migration of the server to larger, more capable hosts as demand dictates likewise is invisible to the client. The only change evident to the client is that the service seems faster.

It is possible applying a standard compression algorithm such as Lempel-Ziff-Huffman to the image slice pixels would also help to boost throughput (see below) given many pixel series consist of runs of minimal variation. However, given the local nature of these algorithms, using compression effectively with the rasterizing performed by the *NtAS *return queue will require considerable experimentation and testing.

Though the XML interface uses a ubiquitious W3C encoding standard to exchange information, we would also like to adapt this protocol to the web service description language (WSDL), so as to make the server more interoperable in a Service Oriented Architectural (SOA) environment. We have developed WSDL services as a part of the NeuroTerrain Image Repository framework [3] and see the utility they can provide, especially for database controlled distribution of binary image data.

### Ease of implementation

The *NT-SDK *is designed to simplify the process of including a *NtAS *client within other Java applications. In fact, the stand alone client application NetOStat distributed via our web site for the Mac OS X, Windows XP, and Linux OS platforms is merely the *NT-SDK *wrapped in a Java source file with a main() function and just a few dozen lines of custom code to instantiate two helper objects (AtlasControllerHelper and SlicerHelper) and implement atlas monitor and driver Java Interface methods. The *NT-SDK *also provides a simple means to simultaneously present multiple axis views of a single atlas data set (e.g., coronal + sagittal), though we've not fully implemented this yet in the distributed NetOStat client. We have also created other custom applications specifically designed to use the NtAS client to jointly browse and analyze other 3D mouse brain data sets aligned to our atlas [2,3], as well as to examine mulitple, aligned atlas data sets in synchrony. In each case, the only code written to provide the NtAS client functionality beyond ~20 lines of API glue code is that needed to add new functionality beyond what the *NT-SDK *already implements.

### Performance and scalability

The use of a processing queue and multiple slicing threads allows the core module of the server to take full advantage of advanced multiprocessor hosts. We have implemented optimization logic on both the server and client side to streamline the image rendering to a series of client knife movement requests over the current Internet. Table 2 provides a brief summary of throughput testing we have done under varying network and operating system conditions. One should note, given the underlying collision-sensitive nature of Ethernet transmission, the additional software layers present between the application and the OS-level TCP/IP stack, and the complex logic associated with opening and controlling communicating sockets make such metrics prone to considerable moment-to-moment variablity; thus, one needs to take variability into account when assessing these metrics. Even though ping latency was measured, the metrics taken are clearly approximate, especially without normalization using an independent measure of network throughput.

**Table 2:** Results of NtAS-NetOstat network throughput results

***Client***	***Server***	***Network***	***Throughput (KB/s)***	***Frame rate (fps)***	***Application Latency (ms)***	***Transmission Time (s)***	***Completed Frames***	***Partial Frames***	***Partial Frame %***	***Ping ***
3GP4-MdkLnx	Loki	1000 Mb/s Smart Switch	6450 +/- 675	8.7 +/- 0.9	1322 +/- 666	0.117 +/- 0.014	1 +/- 0	n.a	n.a.	0.62 +/- 0.88
3GP4-WinXPSP2	Loki	1000 Mb/s Smart Switch	6665 +/- 386	9.0 +/- 0.5	1294 +/- 357	0.112 +/- 0.007	1 +/- 0	n.a.	n.a.	0.0
PBG4	Loki	1000 Mb/s Smart Switch	4060 +/- 757	5.47 +/- 1.02	55.0 +/- 24.2	0.563 +/- 0.067	1.8 +/- 0.48	2.2 +/- 1.8	37.2 +/- 7.7	0.62 +/- 0.48
PBG4	Loki	100 Mb/s Drexel LAN	8530 +/- 131	11.5 +/- 1.75	49.7 +/- 5.77	0.424 +/- 0.070	3 +/- 1	4.7 +/- 1.5	42% +/- 9.8	0.77 +/- 0.55
PBG4	Loki	1.5 Mb/s DSL-East coast	141 +/- 2	0.20 +/- 0.0	103 +/- 32.3	6.17 +/- 0.100	1 +/- 0	8.0 +/- 4.2	2.37 +/- 0.92	27 +/- 15
PBG4	NrtrnBIRN	1.5 Mb/s DSL-East coast	104 +/- 17	0.15 +/- 0.02	238 +/- 122	8.12 +/- 1.36	1 +/- 0	5.8 +/- 1.8	1.89 +/- 0.55	163 +/- 123
PBG4	NrtrnBIRN	100 Mb/s Drexel LAN	752 +/- 116	1.0 +/- 0.16	104 +/- 12.4	1.22 +/- 0.195	1 +/- 0	2.3 +/- 0.58	8.9% +/- 0.44	74 +/- 3.0

20 identical knife slice requests were sent in rapid succession (inter-request interval < 0.1 ms). Requested cross-sections consisted of 576 × 320 (184320 pixels). Were all 20 returned in their entirety to the client, they would consist of ~740 separate 249 pixel rasters. Those rasters returned by the server were not rendered in order to control for client display performance. Metrics for both throughput and latency are the mean of 5 independent tests run ~10 seconds apart. As indicated, not all requested images were returned in full due to the efficiency logic in the server and client. The metrics, based on those pixel rasters returned to the client, are: ***throughput***(total byes/total time excluding application latency), ***frame rate ***(throughput scaled to test image size), **application *latency ***(time in ms from first request to first raster returned), ***transmission time ***(time in seconds from first to last raster returned for all 20 requests), ***completed frames ***(number of complete image raster sets returned), ***partial frames ***(number of incomplete image raster sets returned), ***partial frame % ***(for incomplete images the percent rasters returned), ***ping ***(round-trip latency to server). For all of the tests where client and server were on separate machines, one of two NtAS servers were used (**Loki **at Drexel U., Philadelphia, PA, USA or **NrtrnBIRN **at UCSD, San Diego, CA, USA), each of which had the following configuration: dual 2.3 GHz G5 PPC Macintosh XServe/2.0 GB PC3200 ECC SDRAM (SBus 667 MHz, Cache Bus 1.15 GHz) running Mac OS X 10.3.9. The following clients configurations were tested: **PBG4 **(Macintosh PowerBook 1.5 GHz G4 PPC/1.5 GB PC2700 DDR SDRAM running Mac OS X 10.4.8); **3GP4-MdkLnx **(3.0 GHz Pentium 4/2 GB DDR RAM running Mandrake-Linux v10.1); 3GP4-**WinXPSP2 **(3.0 GHz Pentium 4/1 GB DDR SDRAM running Windows XP SP2 w/2GB VM). All clients machines used the JVM v1.5.0 to run *NetOStat *– for Linux and Windows XP as distributed by Sun Microsystems; for Mac OS X as distributed by Apple Computer. *All tests were performed at 3 AM EST, when both the Internet and LAN activity is at a minimum and is the most stable*.

With those caveats in mind, the following observations can be made: (1) for all machines tested, a standard, university LAN connection (100 Mb/s) provided excellent throughput (>8 MB/s; >11.5 frames/sec); (2) though considerably slower (~0.75 MB/s; 1.0 fps), when connected over the public Internet (DrexelMed LAN to UCSD), performance was acceptible for such an application – ~1.25 seconds to fully complete image transfer for a stream of knife requests typical of that generated via *VK *knife dragging action (20 rapid streamed requests). This is reasonable given typical university-to-university available bandwidth (e.g., OC-1 is ~5 MB/s). On an Internet2 trunk, performance would be much closer to LAN performance given the greatly decreased latency and increased bandwidth (~1 GB/s); (3) throughput over a typical, home Internet connection (DSL, 1.5 Mb/s) whether to a proximal region on the Internet or from East coast (MAE-East) to West coast (MAE-West) was ~3 times slower than when using a typical university-to-university Internet connection and a bit too sluggish for practical use; (4) application latency for Windows and Linux is much longer than on Mac OS X, essentially injecting a 1 second delay prior to image refresh. Given the differences in JVM, OS, and hardware, it will require additional low level testing beyond the scope of this study to determine the root cause of this delay; (5) the algorithmic efficiencies actually make performance fall off a little under very low latency conditions (1000 Mb/s switch). Though this is not a typical runtime scenario, it could be addressed in the future by modulating the communication logic using runtime latency and bandwidth data generated with the throughput/latency testing methods embedded in the *NT-SDK. In summary, as relates to end-user, interactive experience using NetOStat, the total refresh time in response to a typical VK movement is 0.5 and 1.5 seconds for LAN and university Internet connectivity, respectively – quite acceptable for the intended use of the NtAS-NetOStat architecture*.

## Conclusion

The overiding application driving development of this client-server 3D data set analysis environment has been the pressing need within neuroanatomics (*neuroanatomical *informatics) to provide an easy-to-use platform promoting the synergistic use of: (1) brain atlasing systems based on full 3D representation of gray-scale data for both histological[9,24] and MRI[10] brain atlases supporting rapid slicing through the volume in arbitrary planes; (2) existing public terminological respositories for neuroantomical information (e.g., NeuroNames); (3) ontological frameworks with knowledge both of terminology and basic spatial relations (e.g., Foundational Model of Anatomy[26]). The overall goal is to use such a system to support automatic and semi-automatic, meta-analysis of the accumulating large-scale, anatomically-mapped, mouse CNS data sets [5,11,13,14,18-20]. This system is also critical to providing a means for researchers to examine and analyze data acquired on our Cryoplane Fluorscence Microscope (CFM)[27].

The system described here provides a powerful means of displaying atlas and other 3D biomedical image data. It fully supports requirements 1 and 2 above, and we expect to integrate a means to provide for 3 in the future. By moving most of the heavy lifting from the client desktop to a dedicated server and using our high-performance Macrovoxel format on the server side, the data presented may be made more accessible to a wider range of end users. The *NT-SDK *specifically provides a very simple means to combine data from many different NeuroTerrain server hosts, or inclusion of atlas data served by a *NtAS *in a wide range of end-user applications, with little effort on the part of the developer. The value of being able to browse atlas-aligned data sets, in an intuitive way, such as the vast Mouse Brain Library, can not be overstated. Finally, we believe the implementation by NT and other Mouse BIRN atlases of the BIRN Atlas Interoperability API provides an example of how to address the emerging need for such standards to support sharing of biologically relevant segmented geometries derived using a variety of tools from image data sets. This interoperability standard can serve as an interface around which a community atlas mediation service could be constructed.

## Availability and Requirements

*Project name*: NeuroTerrain Atlas Server & NetOStat NeuroTerrain Client;

*Project home page*: ;

*Operating system(s)*: Platform independent;

*Programming language*: NT Atlas Server (ANSI C/C++); NetOStat (Java);

*Other requirements*: Java 1.4.2 or higher required for NetOStat

*Licence*: GNU GPL

Written in Java, the *NT-SDK *has been tested on MacOS X (versions 10.3.x & 10.4.x), Windows XP SP2, and Linux (Mandriva distribution) using Java 1.4.2 and 1.5.0. It is available both as source code and compiled byte-code on an as-is basis. Source code for the NtAS is not currently available, but clients can obtain a server address from the same URL listed above for use with the *NT-SDK *and/or the NetOStat client built on the *NT-SDK*[see also Additional files 1 and 2]. We plan in the future to create a 64-bit Linux cross-compile build file for the server. At the present time we are not planning to distribute the NtAS as source due to the complexity this engenders for further development and distribution of the NT-SDK both in source and compiled bytecode form.

## List of Abbreviations

API Application Programming Interface

BGEM Brain Gene Expression Map located at St. Jude Children's Hospital in Memphis, TN [28]

BIRN Biomedical Informatics Research Network [6]

CFM Cryoplane Fluorescence Microscopy

CSLM Confocal Laser Scanning Microscopy

DM Data Manager – the server module responsible for managing access by the rest of the server components to the image datasets provided by the server application.

GENSAT The Gene Expression Nervous System Atlas[17] sponsored by the NIH National Institute of Neurological Disorders and Stroke (NINDS) which currently includes the BAC Transgenic Project located at The Rockefeller University and BGEM.

GUI Graphical User Interface

JVM Java Virtual Machine

MRM Magnetic Resonance Microscopy

NtAS NeuroTerrain Atlas Server

NT-SDK NeuroTerrain Software Development Kit – the packaged Java framework used by other Java-based software seeking to act as a client to the NeuroTerrain Atlas Server – e.g., an applet, application, servlet, EJB-based application server, etc. It contains pre-built classes with data structures, convenience methods, and Interface-based callbacks greatly facilitating the process of communicating requests to the NT Atlas Server and using the server responses effectively. It also includes an easy to implement JPanel containing both the NT Atlas Client controls and the viewing for atlas-derived data. The NT-SDK-NtAS-Comm portion of the SDK contains only the server communication and control classes. The additional NT-SDK-NetOStat-Gui library provides all the interactive GUI classes seen in the NetOStat application.

OPT Optical Projection Tomography

PQM Process Queue Manager – the server module that provides communication between the server modules and the slicing modules.

SOA Service-Oriented Architecture

SMART Spatial Markup and Rendering Tool – The interactive atlas interface developed for the Mouse BIRN sub-project with BIRN

SV Slice Viewer

VK Virtual Knife

VOI/ROI Volume of Interest (3D)/Region of Interest (2D)

WSDL Web Service Description Language

XML eXtemsible Markup Language – A standardized way of encoding a data stream such that the stream is mostly self-documenting and both machine and human readable.

## Authors' contributions

Author CG developed the binary communication protocols, wrote the server and created an early prototype of the Java-based atlas client.

WB wrote NT-SDK and assisted in debugging the communication protocols.

JN guided the project and provided overall direction and focus.

## Supplementary Material

Additional File 1 NetOStat for Mac OSX. This Mac OS X disk image file a build of the NetOStat client application best suited for use on the Mac OS X operating system. Click here for file

Additional File 2 NetOStat for Windows XP and Linux. This ZIP compressed archive file includes a build of the NetOStat client application suited for use on the Linux or Windows XP operating systems. Click here for file
